# Editorial: Nutrient Use-Efficiency in Plants: An Integrative Approach

**DOI:** 10.3389/fpls.2020.623976

**Published:** 2020-12-15

**Authors:** Manuel Nieves-Cordones, Francisco Rubio, Guillermo E. Santa-María

**Affiliations:** ^1^Departamento de Nutrición Vegetal, Centro de Edafología y Biología Aplicada del Segura-CSIC, Murcia, Spain; ^2^Instituto Tecnológico Chascomús (INTECH), Consejo Nacional de Investigaciones Científicas y Técnicas, Universidad Nacional de San Martín, Buenos Aires, Argentina

**Keywords:** nutrient use efficiency, plant, uptake, mobilization, abiotic stress, fertilizer

Modern agriculture faces major issues resulting from the need to ensure crop production for a growing population while also minimizing the environmental impact of agricultural practices as well as the cost associated with them, all of which can be worsened by the high incidence of abiotic stresses imposed by climate change. The agricultural use of land for crop production leads to the continuous extraction of soil nutrients. In the absence of adequate nutrient replenishment, a subsequent decrease of soil nutrient availability may occur, exerting a negative effect on yield. Yield limitation could also occur as a consequence of there being intrinsically low levels of nutrients in soils. Strategies to overcome low nutrient availability in soils mainly rely on the use of fertilizers and crop breeding. According to the Food and Agricultural Organization (FAO, 2019), approximately 109 Mt of nitrogen (N), 45 Mt of phosphorus (P), and 38 Mt of potassium (K^+^) were used during 2017. Some of the fertilizers applied are based on the use of rock reservoirs that constitute a non-renewable resource. In addition, the use of fertilizers can result in collateral problems, as they can harm ecosystems, with additional costs to agricultural practices. This is a major drawback for low input agriculture as practiced in some regions. In this complex context, ensuring sustainable agriculture requires the adoption of innovative strategies that involve new concepts in the management of agroecosystems, including the use of bio-fertilizers, and the genetic improvement of crops and pastures. One promising goal is to develop crops with high nutrient use efficiency and high resilience to environmental constraints.

Nutrient use efficiency is typically divided into two interactive components: the efficiency of nutrient acquisition (i.e., the amount of nutrient taken up by plants in relation to nutrient supply) and the efficiency of nutrient utilization, which informs the biomass produced by the unit of nutrient incorporated by plants. The existence of relevant constraints in improving these components while maintaining food quality has been highlighted (Barraclough et al., 2010). Further knowledge of the genetic basis, as well as the physiological and molecular mechanisms that determine these efficiencies in plants, may allow for the design of original approaches to addressing these constraints. This Research Topic from Frontiers in Plant Science includes contributions made by several authors from different countries to help to illuminate some of the specific aspects involved in nutrient use efficiency in plants.

N, P, and K^+^ constitute the most common nutrients in fertilizers. To optimize and reduce the applications of fertilizers, we require more insight into how these nutrients are taken up and utilized by plants. With respect to N, four research articles cover aspects related to N use efficiency (NUE) at different levels. Two of them deal with grain yield. Paponov et al. compared two maize varieties with contrasting NUEs. The authors found evidence suggesting that kernel set was differentially regulated in these genotypes, and propose that they play a major role in sink restriction. In turn, Fan et al. evaluated the consequences of manipulating a component of the autophagy machinery, OsATG8b. Autophagy constitutes a relevant process in nutrient use-efficiency at the reproductive stage. Increased expression of *OsATG8b* increased yield, grain quality, and higher transfer of N to the seeds.

A different way of improving NUE in plants could be based on chloride (Cl^−^) supply, as shown by Rosales et al.. Taking into account the fact that, as is the case for nitrate (${\text{NO}}_{3}^{-}$), Cl^−^ fulfills osmotic functions in the vacuole, the authors showed that Cl^−^ enables a higher amount of ${\text{NO}}_{3}^{-}$ to be available for assimilation. Indeed, high Cl^−^ supply specifically increased N Utilization Efficiency (NUtE) with little or no effect on N uptake. The positive effects of Cl^−^ supply on NUE were also observed in most of the plant species examined. Concerning ${\text{NO}}_{3}^{-}$ and Cl^−^ nutrition, Rubio et al. address the impact of high carbon dioxide (CO_2_) and high bicarbonate (${\text{HCO}}_{3}^{-}$) on cytosolic ${\text{NO}}_{3}^{-}$ and Cl^−^ pools in leaves of *Posidonia oceanica* by using ion-sensitive microelectrodes. Importantly, the current rise in atmospheric CO_2_ levels may affect nutrient accumulation by crops (Loladze, 2014). Elevated CO_2_ results in higher levels of ${\text{HCO}}_{3}^{-}$ in seawater, which is used by aquatic plants as a carbon source for photosynthesis. The studies on *Posidonia* by Rubio et al. showed that the high cytosolic ${\text{HCO}}_{3}^{-}$ together with a concomitant increase in cytosolic pH, activated S-type anion channels which allowed ${\text{NO}}_{3}^{-}$ and Cl^−^ efflux from leaf cells. Thus, the effect of high atmospheric CO_2_ on ${\text{NO}}_{3}^{-}$ and Cl^−^ retention in *Posidonia* deserves further investigation, particularly regarding how it could affect NUE.

The efficient use of nutrients by plants requires tight regulation of the systems involved in their homeostasis. Increasing evidence points to a co-regulation among the homeostasis of different nutrients. Raddatz et al. examined the interactions between K^+^ and ${\text{NO}}_{3}^{-}$ nutrition. In addition to describing uptake and distribution systems, the authors highlighted how these processes were co-regulated. Important components of this co-regulation are the CIPK23/CBL complex—which post-translationally regulates both K^+^ and ${\text{NO}}_{3}^{-}$ transporters by their phosphorylation—and the existence of transporters, such as NRT1.5, with the ability to transport both macronutrients. In addition, the interactions of sodium (Na^+^) with K^+^ and ${\text{NO}}_{3}^{-}$ nutrition were also covered because of the important effects exerted by the former on the nutrition of the others. In turn, the review by Villette et al. was centered on the transport systems that regulate the K^+^ concentration of grapevine berries, which is critical for the optimal quality of fruits and wine properties. Special attention was given to the detrimental effects that climate change will have on these parameters. In particular, high temperatures lead to an increase in K^+^ and sugar accumulation in berries, which results in a lower quality of wine.

Two papers cover P use efficiency (PUE) at different levels. Galatro et al. present a review article about the role of nitric oxide (NO) under P-deficient conditions. NO is a signaling molecule associated with N metabolism. The authors gave an update on the acclimation responses to low P and the NO signaling pathways in plants. The authors stated that there is a potential to improve P-deficiency responses in plants by understanding the contribution of NO on this topic. A second review on P, by Powers and Thavarajah, deals with the PUE of field pea. This leguminous crop has high protein/micronutrient content but it is not widely cultivated. Due to this high content, field pea could be used to replenish N levels in soils after culture rotation. However, it has a high P demand. The authors hypothesize that diversity may exist among field pea varieties regarding PUE and encourage further exploration of its natural diversity to enable potential cultivation.

Regarding magnesium (Mg^2+^) nutrition, Ogura et al. described Mg^2+^ deficiency and its effects on the retranslocation of other mineral nutrients within the plant as well as changes in sucrose partitioning, photosynthesis, and biomass production. These observations were in agreement with the described crosstalk in the nutrition of different nutrients (Amtmann and Blatt, 2009; Kellermeier et al., 2014). A second contribution on Mg^2+^ by Wang et al., consists of a meta-analysis from 99 field research articles to determine the effect of Mg^2+^ fertilization on crop yield and other agronomic parameters.

Regarding micronutrients, Alejandro et al. contribute a comprehensive review of the systems involved in manganese (Mn) uptake and compartmentalization. Most of the proteins that transport Mn are not specific for the metal and can transport other divalent cations. Thus, diverse families of transporters are involved in the Mn homeostatic network. Dimkpa et al. present an original research paper on an alternative and more-efficient approach to zinc (Zn) fertilization which enhances Zn uptake under drought stress in wheat plants. By supplying Zn as nano-particles, increases in plant performance and Zn accumulation were achieved.

Avoiding high levels of toxic elements is critical in modern agriculture. Adams et al. contribute an original research paper showing that sulfur supply alleviated aerial chlorosis and growth retardation caused by cesium (Cs^+^) stress without reducing Cs^+^ accumulation in Arabidopsis plants. Their findings indicate that exposure to Cs^+^ increased glutathione accumulation, a process that can be promoted by the exogenous application of sulfur-containing compounds.

The regulation of the transport proteins is a key process of nutrient homeostasis. A review by Saito and Uozumi examines how plants control the uptake and balance of nutrients by Calcium-regulated phosphorylation, a mechanism emerging as pivotal for the regulation of transport systems. The review is focused on CPK and CBL-CIPK systems, the latter also highlighted by Raddatz et al.. These regulatory systems are crucial for maintaining important physiological processes such as intracellular osmolality, cell expansion and movement, salt stress tolerance, the control of ammonium (${\text{NH}}_{4}^{+}$) uptake, regulation of metal ions uptake, and toxins.

Lastly, Dreyer and Michard offer an original research article that challenges the classical concept of high-and low-affinity uptake systems. The authors propose that the affinity concept based on enzyme kinetics did not have proper scientific grounds. Using a simulation-based approach, the authors showed that affinities for substrate transport may be an artifact of data display, or that the transport affinities originated from the voltage- and pH-dependency of the H^+^-ATPase. Thus, the classification of transporters based on these studies is misleading thermodynamically.

Collectively, the contributions to this Research Topic provide very interesting and relevant information on the homeostasis of mineral nutrients in plants and its effects on dry matter production and grain quality. The articles cover important aspects such as the systems involved in nutrient acquisition and utilization, the components involved in their regulation, and the existence of common regulatory pieces that support the observed cross-talk in the homeostasis of different nutrients. The information gathered in this Research Topic could be used to develop tools for improving the nutrient use efficiency of plants, aiming at generating crops better suited for future agriculture.

## Author Contributions

The editorial was written, corrected, and accepted by all authors.

## Conflict of Interest

The authors declare that the research was conducted in the absence of any commercial or financial relationships that could be construed as a potential conflict of interest.
